# Protective autophagy by thymidine causes resistance to rapamycin in colorectal cancer cells *in vitro*

**DOI:** 10.20517/cdr.2021.21

**Published:** 2021-05-24

**Authors:** I.V. Bijnsdorp, Godefridus J. Peters

**Affiliations:** ^1^Department of Medical Oncology, Amsterdam UMC, location VUMC, 1007 MB Amsterdam, Netherlands.; ^2^Department of Urology, Amsterdam UMC, location VUMC, 1007 MB Amsterdam, Netherlands.; ^3^Department of Biochemistry, Medical University of Gdansk, 80-210 Gdansk, Poland.

**Keywords:** Thymidine phosphorylase, mTOR, rapamycin, thymidine, thymidine phosphorylase inhibitor, autophagy

## Abstract

**Aim:** Thynidine phosphorylase (TP) acts as a proangiogenic growth factor which may regulate mammalian Target of Rapamycin (mTOR). We investigated whether the TP substrate thymidine and overexpression of TP affected mTOR signaling by comparing Colo320 (TP deficient) cells and its TP-transfected variant (Colo320TP1).

**Methods:** Drug resistance was assessed with the sulforhodamine B assay, protein expression with Western blotting, cell cycle distribution and cell death with Fluorescence-activated cell sorting analysis, and autophagy with immunofluorescence.

**Results:** Colo320 and Colo320TP1 cells had comparable levels of sensitivity to the mTOR inhibitor rapamycin. Thymidine treatment led to 13- and 50-fold resistance to rapamycin in Colo320 and Colo320TP1 cells, respectively. In Colo320TP1 cells, the thymidine phosphorylase inhibitor (TPI) reversed the thymidine induced resistance to rapamycin, but not in Colo320 cells, indicating a role for TP in the protection. Thymidine increased p70/S6k-phosphorylation (downstream of mTOR) in Colo320TP1, but it was not affected in Colo320. As a mechanism behind resistance, we studied the levels of autophagy and found that, in Colo320TP1 cells, autophagy was highly induced by thymidine-rapamycin, which was decreased by TPI. In addition, the autophagy inhibitor 3-methyl-adenine completely inhibited autophagy and its protection.

**Conclusion:** Rapamycin resistance in TP-expressing cancer cells may therefore be related to thymidine-mediated autophagy activation.

## INTRODUCTION

Thymidine phosphorylase (TP) is also known as platelet derived-endothelial cell growth factor, which plays a role in angiogenesis^[1]^. TP is often overexpressed in cancer, including colorectal, breast and bladder cancer^[2]^. When upregulated, TP is related to a poor prognosis for the patient, induces metastasis, and leads to a higher microvessel density^[3,4]^. The enzymatic activity of TP has been demonstrated to be essential for stimulating angiogenesis in various studies^[5]^. Therefore, inhibitors of TP have been developed as anti-angiogenesis therapy, including the thymidine phosphorylase inhibitor (TPI)^[6]^. TPI inhibits TP very potently and specifically and has demonstrated anti-angiogenic activity. However, its primary application is in the combination with trifluorothymidine (TFT) in TAS-102 (Lonsurf), which is registered as third line therapy in the treatment of advanced colorectal cancer^[7,8]^.

It is not clear whether angiogenesis plays a role in the efficacy of TAS-102^[9]^. However, various studies clearly indicated a role of TP in angiogenesis, although the exact mechanism behind TP-mediated angiogenesis remains unclear. TP converts thymidine (TdR) to thymine and deoxyribose-1-phosphate (dR-1-P)^[10]^. dR-1-P can be converted to deoxyribose-5-phosphate or deoxyribose (dR). dR is often thought to be the main regulator behind TP-mediated angiogenesis because it is the main product that can be secreted by the cells^[10]^. Moreover, dR was demonstrated to be pro-angiogenic in various *in vitro* and *in vivo* studies^[11-16]^. In addition to dR, various angiogenic factors may also be involved in the anti-angiogenic effects of TP, such as Interleukin-8 (IL-8) and Vascular Endothelial Growth Factor^[17-19]^.

Several mechanistic studies on TP-mediated angiogenesis indicated that p70/S6k phosphorylation was involved in migration and invasion^[11,20]^. P70/S6k is a kinase that is directly downstream of mTOR (mammalian target of rapamycin) and plays a role in signaling to proliferation and angiogenesis^[21]^. Various inhibitors of mTOR have been developed, next to rapamycin, which is an immunosuppressant and is used to prevent rejection after organ transplantation. Since it has strong antiproliferative activity, rapamycin and other potent novel analogs (temsirolimus and everolimus) have been evaluated for their anticancer effects. Both drugs have been registered for treatment of renal cell cancer. mTOR now represents an attractive anti-tumor target, either alone or in combination^[21,22]^. One of the mechanisms of action of rapamycin is the induction of autophagy. Autophagy may result in the induction of cell death, but it can also cause protection against cell death^[23]^. Autophagy may also provide protection against cytotoxicity induced by 5-fluorouracil (5-FU), but not by TFT^[24-26]^. Inhibition of autophagy with 3-methyl-adenine (3-MA) enhanced the sensitivity to 5-FU. In addition, for other anticancer drugs, it has been demonstrated that autophagy may protect against their action, which can be reversed by inhibitors such as (hydroxy)chloroquine^[27]^. Several clinical studies have been initiated to reverse resistance due to autophagy protection^[28,29]^. Therefore, autophagy appears to be an interesting target to increase chemosensitivity.

To determine to what extent TP plays a role in the p70/S6k signaling pathway, Colo320 and Colo320TP1 cancer cells (deficient and with high *TP *expression, respectively) were exposed to rapamycin, combined with TdR and TPI. In the present study, we show that TdR addition results in rapamycin resistance. Downstream effects of mTOR and autophagy may play a role in this protective effect. These data provide important information for the application of the mTOR inhibitors in the clinic.

## METHODS

### Cell culture and chemicals

The human colon carcinoma cell line Colo320 was obtained from the American Type Culture Collection and Colo320TP1 was transfected with TP, as described previously^[30]^. Cells were cultured as monolayers in Dulbecco’s Modified Essential Medium supplemented with 10% heat inactivated fetal calf serum and 20 mM Hepes in 25 cm^2^ culture flasks (Greiner Bio-One, Frickenhausen, Germany). Cells were maintained in a humidified 5% CO_2_ atmosphere at 37 °C. TPI was provided by Taiho Pharmaceuticals Co. Ltd. (Tokushima, Japan). 3-Methyladenine (3-MA) and TdR were obtained from Sigma Aldrich Chemicals (Zwijndrecht, The Netherlands). TdR was dissolved in phosphate buffered saline (PBS) in stock solutions of 20 mM.

### Drug cytotoxicity assays

Drug cytotoxicity was determined by the sulforhodamine B (SRB)-assay^[31]^. Two thousand cells/well were seeded in 96-well plates (Greiner Bio-One). After 24 h, enabling attachment, cells were exposed to increasing concentrations of rapamycin for 72 h, with and without 100 µM TdR and/or 10 µM TPI. For experiments where 3-MA was used, cells were exposed to 10 mM 3-MA^[17]^ simultaneously with all tested combinations. After 72 h of drug exposure, cells were precipitated with trichloroacetic acid for 1 h at 4 °C, colored with SRB and analyzed as described previously^[31]^. To calculate the growth inhibition curves, optical density values were corrected for readings on the day of drug addition. The 50% growth inhibitory concentration (IC_50_) values were subsequently determined from graphs and are given as means ± SEM.

### Fluorescence-activated cell sorting analysis of cell cycle distribution

Cell cycle analysis and apoptosis measurements were performed as described previously^[32]^. In brief, 200,000 cells were seeded in six-well plates. After 72 h treatment, cells were trypsinized, resuspended in medium that was collected from the matching sample and centrifuged for 5 min at 1200 rpm. Subsequently, cells were stained with propidium iodide (PI) buffer (0.1 mg/mL with 0.1 % RNAse A) in dark on ice. DNA content of the cells was analyzed by Fluorescence-activated cell sorting (FACS) (Becton Dickinson) with an acquisition of 10,000 events. The sub-G1 peak was used to determine the extent of cell death.

### Western blotting

Colo320 cells were seeded at 1.5 × 10^6^ in 25 cm^2^ culture flasks (Greiner Bio-One). After 6 h, cells were exposed to 100 µM TdR, 10 µM TPI, or 20 nM rapamycin. After treatment, cells were scraped in lysis buffer (Cell Signaling Technology, Inc, Danvers, MA, USA) supplemented with 0.04% protease inhibitor cocktail and centrifuged at 14,000 rpm at 4 °C for 10 min. Protein concentration in the supernatant was determined by performing a Bio-Rad protein assay according to the manufacturer’s instructions (#500-0006, Bio-Rad Laboratories, Veenendaal, The Netherlands). From each condition, 30 µg of protein were separated on an 8%-10% SDS-PAGE gel and electroblotted onto a fluorescence polyvinylidene fluoride-membrane (Millipore Corp., Billerica, MA). Blotted membranes were blocked in Rockland blocking buffer (Rockland, Tebu-bio, Heerhugowaard, The Netherlands) for 1 h at room temperature (RT) and subsequently incubated overnight at 4 °C with the primary antibodies directed against Akt (#9272), phospho-Akt, (Ser473 #9271), p70/S6k (#2708), phospho-p70/S6k (#9205), mTOR (#2983), and phospho-mTOR (#2971) (1:1000; Cell Signaling Technology) in the Rockland blocking buffer and PBS-Tween solution (0.05% Tween-20). Antibody targeted against β-Actin (A5441) was used at a 1:10,000 dilution. The membranes were washed five times in PBS-Tween and incubated with the secondary infrared labeled antibody (1:10,000) for 1 h at RT in the dark. After incubation, the membrane was washed in PBS-Tween and then 5 min in PBS without Tween-20 to reduce the background. Subsequently, the bands were scanned using Odyssey Infrared Imager, with the settings: 84-µm resolution, 0-mm offset, and high quality^[10]^.

### Immunofluorescent staining of autophagic vesicles

Immunofluorescent staining was performed as described previously^[24,32]^. In brief, cells were seeded in six-well plates on a cover slip and exposed to rapamycin, TPI, and/or TdR for 72 h. After exposure, cells were fixed for 15 min in 4% formaldehyde and permeabilized with methanol for 10 min at -20 °C. Subsequently, cells were blocked and the LC3B antibody was added (1:200, Cell Signaling; overnight at 4 °C), after which the secondary antibody goat-anti-rabbit conjugated with Alexa Fluor was added together with Hoechst 33342 (1:1000) for 1 h at RT. The coverslips were mounted onto microscope slides using Vectashield (Vector, Burlingame, CA, USA). Fluorescence microscopy was carried out using an inverted Leica DMIRB/E fluorescence microscope (Leica Cambridge, Cambridge, UK). Images were collected using Q500MC Quantimet software V01.01 (Leica Cambridge).

## RESULTS

### Rapamycin cytotoxicity is decreased by TdR

Colo320 and Colo320TP1 had comparable levels of sensitivity to rapamycin, as determined by the SRB-assay [Table 1]. TPI addition to rapamycin hardly affected the sensitivity to rapamycin in both Colo320 and Colo320TP1 cells. However, TdR addition protected both the Colo320 and Colo320TP1 cells against the cytotoxicity induced by rapamycin, which resulted in 13.3-fold resistance in Colo320 but even 50-fold resistance in Colo320TP1 cells. To determine whether this increase was related to the breakdown of TdR by TP, TPI was added to this combination which has been previously been reported to effectively inhibit this conversion^[7]^. As expected, in Colo320 cells (which do not express TP), TPI addition to rapamycin and TdR did not affect the resistance, indicating that in Colo320 cells protection is related to anabolism of TdR to TdR nucleotides. In Colo320TP1 cells, TPI addition to rapamycin and TdR completely reversed the protective effect, indicating a role for TP in the protection.

**Table 1:** Induction of resistance to rapamycin by TdR and its reversal by TPI

	**Rapa**	**Rapa + TPI**	**Rapa + TdR**	**Rapa + TdR+TPI**
**Colo320 - 3-MA**	7.0 ± 1.5	5.0 ± 1.2	93.0 ± 7.0	101.7 ± 1.82
**Colo320 + 3-MA**	5.3 ± 2.1	3.1 ± 0.1	99.0 ± 0.63	105.2 ± 3.16
**Colo320 TP1 - 3-MA**	7.2 ± 1.5	8.8 ± 2.1	355.0 ± 4.2	7.5 ± 1.1
**Colo320 TP1 + 3-MA**	4.1 ± 1.9	1.5 ±0.3	11.5 ± 5.4	11.1 ± 2.5

Cells were exposed to the indicated drugs for 72 h. Values (in nM) represent the mean IC_50_ values of at least three independent experiments ± SEM. Drugs were added simultaneously. Rapa: Rapamycin; TdR: thymidine; TPI: thymidine phosphorylase inhibitor.

### Effects on cell cycle and cell death

**Figure 1:**
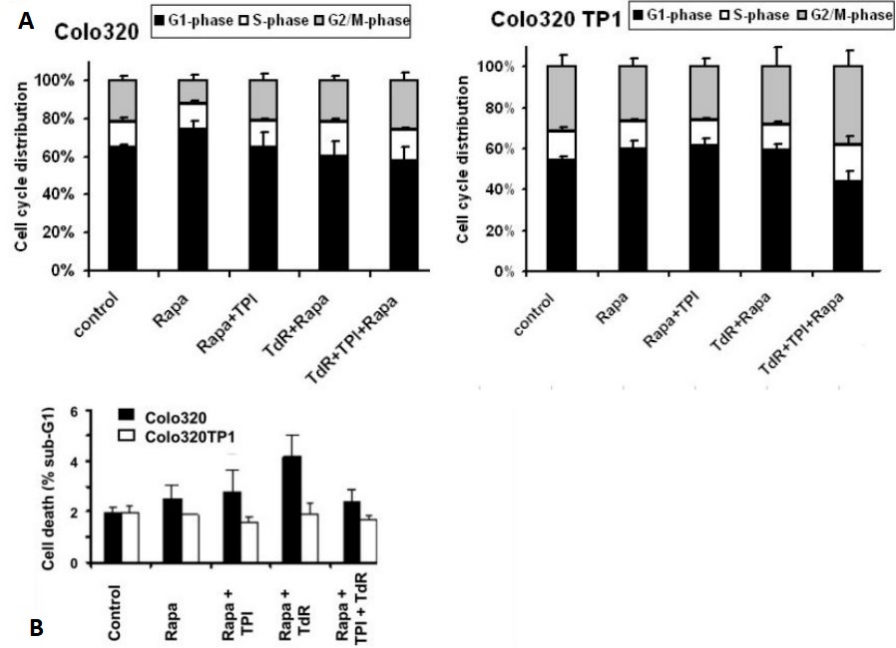
FACS analysis of Colo230 and Colo320TP1 cells after 72 h exposure to 20 nM rapamycin, combined with 100 µM thymidine (TdR), 10 µM TPI, or TdR + TPI: (A) cell distribution; and (B) the percentage of cells in the sub-G1-phase. Values represent means of at least four independent experiments ± SEM. Rapa: Rapamycin; TdR: thymidine; TPI: thymidine phosphorylase inhibitor.

To determine whether the protective effect of TdR was related to specific cell cycle effects, FACS analysis of PI-stained cells was performed. To study this, 20 and 200 nM rapamycin were used, 20 nM being a concentration slightly above that inducing 50% cell growth inhibition, but lower than the IC_50_ concentration of rapamycin + TdR in Colo320TP1 cells, while 200 nM is a concentration causing complete growth inhibition [Table 1]. In both cell lines, 20 nM rapamycin did not significantly affect the cell cycle [Figure 1A], although the G1-phase was slightly increased after rapamycin exposure. TdR addition to rapamycin slightly increased the S- and G2/M-phases in Colo320 cells, which might be related to activation of TdR by thymidine kinase. In Colo320TP1 cells, addition of both TdR and TPI to rapamycin increased accumulation of cells in the S- and G2/M-phase. At 200 nM rapamycin alone (data not shown), the effects on cell cycle distribution in both Colo320 and Colo320TP1 cells were comparable to that at 20 nM. In addition, the combinations showed a similar effect in Colo320 cells, but, in Colo320TP1 cells, the combinations of 200 nM rapamycin with TPI and TdR increased the fraction of cells in S-phase to 20% and 25%, respectively, with a similar effect on the G2/M-phase (25%). In the triple combination of rapamycin, TPI, and TdR, the effect on the S-phase was around 25%, but that on the G2/M-phase increased to 40%.

To determine whether the protective effect against rapamycin was mediated by a decreased cell death induction, we analyzed the sub-G1-fraction of PI-stained cells at 20 nM rapamycin. As expected, the effect of 20 nM rapamycin was limited and only present in Colo320 cells and absent in Colo320TP1 cells [Figure 1B]. In Colo320 cells, the addition of TdR increased cell kill, but not significantly, while addition of TPI reduced the cell death induction.

### Effects of TdR, TPI and rapamycin on intracellular kinase phosphorylation levels

**Figure 2:**
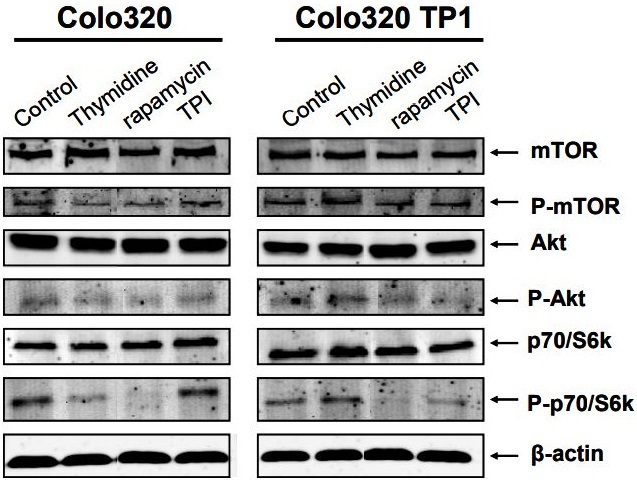
Western blot of expression levels of intracellular protein kinases after 6 h exposure to 100 µM thymidine (TdR), 20 nM rapamycin, or 10 µM TPI. Blot is representative of three independent experiments. TPI: Thymidine phosphorylase inhibitor.

Rapamycin is a specific inhibitor of mTOR, a serine-threonine protein kinase directly downstream of Akt and upstream of p70S6k. Therefore, the expression and phosphorylation levels of these intracellular kinases were determined. In Colo320 cells, TdR and rapamycin decreased phosphorylation of mTOR, Akt, and p70S6k [Figure 2], but TPI increased the phosphorylation of p70/S6k. In Colo320TP1 cells, TdR increased p70S6k phosphorylation slightly. Rapamycin completely inhibited p70/S6k phosphorylation levels, while mTOR phosphorylation hardly decreased. TPI alone hardly affected the intracellular signal transduction pathway in these cells. The protective factor of TdR in Colo320TP1 cells was possibly mediated by activation of p70/S6k by TdR, while, in Colo320 cells, this seems to be mediated by another mechanism.

### Effects of TdR, TPI and rapamycin on *LC3B* expression in autophagosomes

**Figure 3:**
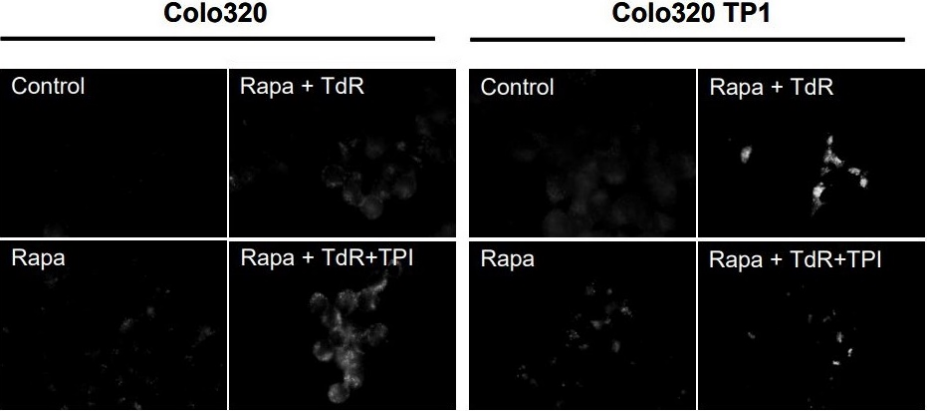
Immunofluorescent staining of LC3B in autophagic vesicles. Cells were exposed for 72 h to 20 nM rapamycin (rapa) and combinations with 100 µM TdR and/or 10 µM TPI. The figure is representative for three independent experiments. Rapa: Rapamycin; TdR: thymidine; TPI: thymidine phosphorylase inhibitor.

Autophagy has been linked to both cell death and protection against cytotoxicity^[23]^. Rapamycin has been reported to kill the cell by inducing autophagy, also known as autophagic cell death. Therefore, we investigated rapamycin induced autophagy, and whether TdR affected this. However, rapamycin hardly induced the formation of autophagic vesicles in both Colo320 and Colo320TP1 cells [Figure 3], but this was slightly increased by TdR in Colo320 cells and highly increased in Colo320TP1 cells. The addition of TPI to rapamycin and TdR increased the number of autophagic vesicles in Colo320 cells. In Colo320TP1 cells, however, TPI addition to rapamycin and TdR decreased the induced autophagic vesicles compared to Rapa + TdR. In summary, TdR addition to rapamycin increased the induction of autophagic vesicles, which was decreased by adding TPI. This indicates a role of TP in the induction of protective autophagy.

### Protection of TdR against rapamycin was mediated by activating protective autophagy

To determine whether the induced autophagy was responsible for the resistance, the autophagy inhibitor 3-MA was added. 3-MA did not affect the sensitivity of Colo320 cells to rapamycin, in both the presence and absence of TdR, rapamycin, and/or TPI, indicating that autophagy was not the mechanism of cell growth inhibition in these cells [Table 1]. This is in agreement with the lack of formation of autophagic vesicles [Figure 3]. However, in Colo320TP1 cells, 3-MA reversed the induced protection by TdR almost completely, showing that autophagy was responsible for the protection against rapamycin cytotoxicity. 3-MA did not change the sensitivity of cells for any other drug combinations.

## DISCUSSION

In the present study, we demonstrated the novel finding that TdR can cause resistance against rapamycin by the induction of protective autophagy. TdR was previously reported to protect against the cytotoxicity of nucleoside analogs such as 5-FU and TFT^[33,34]^ and antifolates^[35]^ but, to our knowledge, not against the cytotoxicity of protein kinase inhibitors. The protective effect was mediated by the induction of protective autophagy.

Rapamycin inhibits the activation (phosphorylation) of mTOR. mTOR signaling results in the activation of various transcription factors that are involved in cell proliferation and angiogenesis. The direct downstream kinase of mTOR is p70/S6k, which was previously reported to be involved in the angiogenic effects of TP^[11,20]^. To study the role of TP in the p70/S6k signaling pathway, we modulated TP activity by addition of TdR and TPI to two cell lines, one with a TP overexpression. We found an almost complete resistance to rapamycin when TdR was added to TP overexpressing cells, which could be completely reversed by addition of TPI. This indicates that TP is essential for the protective effect and that this effect lays downstream of TP.

Since mTOR inhibition has many downstream consequences, the exact molecular mechanism that induced the cytotoxic response in these cells could not be defined completely. However, it seems that, in our models, autophagy is not involved in the cytotoxic effect at the concentration of 20 nM. However, at 200 nM rapamycin, 30%-35% cell kill was observed in both cell lines (data not shown), but the role of autophagy was not investigated. Autophagy has been associated with the effect of rapamycin in various cancer cell types. Autophagy is a catabolic process that is activated by metabolic stress and is induced after cleavage of the light chain 3 proteins in autophagic vesicles^[23,36]^. Autophagy is described as a tumor suppressor mechanism by killing cells undergoing transformation, but it also enables cell survival after the induction of stress^[23,36]^. Hence, autophagy can also act as protection against the cytotoxicity of several anticancer drugs such as 5-FU, gemcitabine, temozolomide, oxaliplatin, and radiation, but not TFT^[24-28]^. It has also been demonstrated that antiviral thymidine analogs can inhibit autophagy as well^[37]^. Other studies focused on the development of autophagy inhibitors to prevent a survival rescue response^[38]^. The exact role of autophagy in cell death and survival is still a subject of debate. In Colo320TP1 cells, cellular protection by TdR was clearly mediated by autophagy, since 3-MA reversed autophagy and TdR protection. We previously observed a similar effect with deoxyribose^[39]^, indicating that the effect of TdR is mediated by this metabolite. This mechanism is different from that in Colo320 cells that do not express TP. This indicates that the protective effect of TdR in these cells is probably related to metabolic activation of TdR by thymidine kinase to its monophosphate (dTMP), after which it can be incorporated into the DNA, stimulating cell proliferation, as seen by the increase in G2/M- and S-phase compared to the effect of rapamycin alone. The data indicate that the use of rapamycin in tumors with a high TdR content may be contraindicated, but they also demonstrate that a combination with TPI would prevent such an effect. Therefore, it is of interest to explore combinations of TAS-102 (which contains TPI) with rapamycin, which should include mechanistic studies evaluating, e.g., the phosphorylation status of Akt, mTOR, and p70/S6k.

In conclusion, resistance to rapamycin may be related to the activation of autophagy by TdR. To optimize the use of mTOR inhibitors, it seems important to study the levels of TdR combined with intratumoral *TP* expression.

## DECLARATIONS

### Copyright

© The Author(s) 2021.
